# A multi-degree-of-freedom reconfigurable ankle rehabilitation robot with adjustable workspace for post-stroke lower limb ankle rehabilitation

**DOI:** 10.3389/fbioe.2023.1323645

**Published:** 2023-11-24

**Authors:** Qingyun Meng, Guanxin Liu, Xin Xu, Qiaoling Meng, Liang Qin, Hongliu Yu

**Affiliations:** ^1^ College of Medical Instruments, Shanghai University of Medicine and Health Sciences, Shanghai, China; ^2^ Institute of Rehabilitation Engineering and Technology, University of Shanghai for Science and Technology, Shanghai, China; ^3^ Shanghai Engineering Research Center of Assistive Devices, Shanghai, China

**Keywords:** rehabilitation robot, rehabilitation training, reconfigurable mechanism, ankle rehabilitation, variable workspace

## Abstract

**Introduction:** A multi-degree-of-freedom ankle rehabilitation robot with an adjustable workspace has been designed to facilitate ankle joint rehabilitation training. It features a rotation center adapted to the human body, making it suitable for patients with ankle dysfunction following a stroke.

**Method:** In this study, a multi-degree-of-freedom reconfigurable ankle rehabilitation robot (RARR) with adaptable features, based on the principles of ergonomics, has been proposed to cater to the varying needs of patients. This robot offers an adjustable workspace, allowing for different types of ankle joint rehabilitation exercises to be performed. By adjusting the assembly of the RARR, personalized and targeted training can be provided to patients, circumventing issues of redundancy in degrees of freedom during its use. A kinematic model of the robot has been established, and finite element simulation has been employed to analyze the strength of critical components, ensuring the safety of the robot. An experimental platform has been set up to assess the smoothness of the rehabilitation process with RARR, with angle measurements conducted using an Inertial Measurement Unit (IMU).

**Results and discussion:** In conclusion, both simulation and experimental results demonstrate that the robot offers an adjustable workspace and exhibits relatively smooth motion, thereby confirming the safety and effectiveness of the robot. These outcomes align with the intended design goals, facilitating ankle joint rehabilitation and advancing the field of reconfigurable robotics. The RARR boasts a compact structure and portability, making it suitable for various usage scenarios. It is easily deployable for at-home use by patients and holds practical application value for wider adoption in rehabilitation settings.

## 1 Introduction

Stroke is an acute cerebrovascular disorder characterized by a high incidence rate, elevated mortality, and a range of complications (Feigin et al., 2021; Markus, 2022). In recent years, the issue of post-stroke motor dysfunction has become increasingly pressing due to the growing number of stroke patients, with data indicating that approximately 65% of survivors require rehabilitation (Miao et al., 2023). Traditional rehabilitation is labor-intensive, and associated with limitations such as the inability to quantify rehabilitation data, strained medical resources, inconsistent training, and high costs (Gittler and Davis, 2018; Gao et al., 2023). Robot-assisted therapy has emerged as a solution to address these challenges. As the fields of rehabilitation medicine and robotics continue to integrate, various models of ankle rehabilitation robots have been designed to address ankle joint dysfunction resulting from strokes (Dao et al., 2022; Meng et al., 2023a). These diverse rehabilitation robots possess unique potential, offering the promise of more effective and consistent rehabilitation solutions for these patients, thereby positively impacting their quality of life and rehabilitation outcomes.

Ankle rehabilitation robots have extensive applications in the field of rehabilitation medicine, including but not limited to ankle injury rehabilitation, athlete rehabilitation, and elderly population rehabilitation, among other application contexts. Traditionally, ankle rehabilitation robots can be categorized into two main types: wearable and platform-based systems (Jiang et al., 2019; Li et al., 2020). Platform-based ankle rehabilitation robots are fixed devices consisting of a base and a footplate. The base incorporates control and power systems, while the patient places their foot on the footplate. The robot then moves the footplate within predefined parameters, assisting patients in recovering ankle joint strength and flexibility. Zou et al. (2022) proposed a 3-RRS (where R and S denote revolute and spherical joints, respectively) parallel ankle rehabilitation robot (PARR) with low mobility parallel mechanisms. Tsoi et al. (2009) presented an approach that aligns the rotation center by using the patient’s ankle as a part of the robot’s kinematic constraints, including the selection of four linear actuators to control platform tilt. However, this approach may inadvertently impose unexpected loads, leading to discomfort and safety concerns. Zhang et al. (2019) developed a parallel ankle rehabilitation robot (PARR) with three rotational degrees of freedom around the virtual stationary center of the ankle joint. They also established a comprehensive information acquisition system to enhance human-machine interaction among the robots, patients, and healthcare providers. These studies emphasize the importance of considering compatibility, convenience, and safety in the design process. Nonetheless, the mentioned rehabilitation devices are often unable to provide targeted treatment solutions to users, leading to unnecessary movements during the rehabilitation process, resulting in redundancy of degrees of freedom. Additionally, their complex structures increase the cost of robot design. To address these issues, Zhang et al. (2017) introduced a redundant-driven reconfigurable robot structure called the Compliance Ankle Rehabilitation Robot (CARR). This robot is powered by four Festo pneumatic muscles, offering an adjustable workspace and actuator torque to meet the range of motion and muscle strengthening requirements for training. However, this rehabilitation equipment is relatively large in size, involves complex setup and adjustments, and has limited applicability in various environments. Furthermore, rehabilitation devices developed using flexible actuators and materials are still in the experimental stage and are primarily found in laboratories (Meng et al., 2023b). Wang et al. (2022) designed a novel reconfigurable ankle rehabilitation exoskeleton capable of static and dynamic rehabilitation exercises and real-time adaptation to the rotation center of the human ankle-foot complex. Yoon and Ryu (2005) developed a reconfigurable ankle rehabilitation robot with multiple rehabilitation modes. This robot can be reconfigured from a range of motion (ROM) or strengthening exercise device by simply adding extra boards to a balance or proprioceptive exercise device.

When developing ankle rehabilitation devices, it is essential to prioritize user needs, such as device adjustability and adaptability, the capability for personalized rehabilitation plans, a comfortable user experience, and low usage costs, among other aspects. However, this presents several challenges: 1. Precision and Safety: Precision and safety are fundamental prerequisites for designing such devices. The robot must ensure the effectiveness and safety of the equipment. 2. Cost and Accessibility: The manufacturing cost of the robot and the accessibility of the equipment pose a challenge. Rehabilitation robots need to be produced within a reasonable price range to ensure widespread availability to medical institutions and patients. 3. Software Development and Algorithm Design: Complex control algorithms need to be developed to enable the robot to provide personalized rehabilitation plans. The causes of ankle injuries in patients are diverse, and each patient may require different rehabilitation approaches. Therefore, the development of a personalized and reconfigurable ankle rehabilitation robot tailored to individual patient needs holds significant importance.

This study presents a multi-degree-of-freedom ankle rehabilitation robot with an adjustable workspace for post-stroke lower limb ankle rehabilitation. The key feature of this robot is its ability to be personalized and assembled according to the specific rehabilitation needs of different users, involving the selection of varying numbers of actuators and assembly modes to achieve different workspaces for the robot. This customization aims to provide personalized treatment, avoid redundancy of degrees of freedom, and alleviate the financial burden on users. The organization of the remainder of this study is as follows: Section 2 provides an overview of the robot’s design, including different assembly modes. Section 3 conducts a theoretical analysis of the robot, including the establishment of the robot’s kinematic model, the construction of the robot’s workspaces in various modes, and finite element simulation analysis to verify the strength of critical robot components, ensuring the robot’s safety and effectiveness. Section 4 establishes an experimental platform and conducts passive control rehabilitation motion experiments with the robot prototype, analyzing the experimental results to validate the feasibility of the robot. Finally, in Section 5, a summary and outlook for this study are provided.

## 2 RARR system design

This section provides a detailed account of the design process for the RARR system, comprising two main aspects: the mechanical structure of RARR and the control system. The mechanical structure can be further subdivided into three key components: reconfigurable design, adjustable design, and rotation center matching design. The RARR’s mechanical structure encompasses three degrees of freedom, and its adaptability is achieved by selecting different numbers of actuators and diverse assembly configurations to accommodate adjustable robot workspaces. These configurations can be set to enable three single-axis motion modes: dorsiflexion/plantarflexion, inversion/eversion, and internal/external rotation; three dual-axis motion modes: dorsiflexion/plantarflexion with inversion/eversion, dorsiflexion/plantarflexion with internal/external rotation, and inversion/eversion with internal/external rotation; and one three-axis motion mode. The adjustable design allows for user-specific adaptability, facilitating both left and right foot interchangeability and catering to users of varying heights. The rotation center matching design is implemented to prevent patients from incurring secondary injuries while using the system. The control architecture of RARR is composed of four main modules: the central control module, sensing module, selection module, and actuation module.

### 2.1 Mechanical design

**FIGURE 1:**
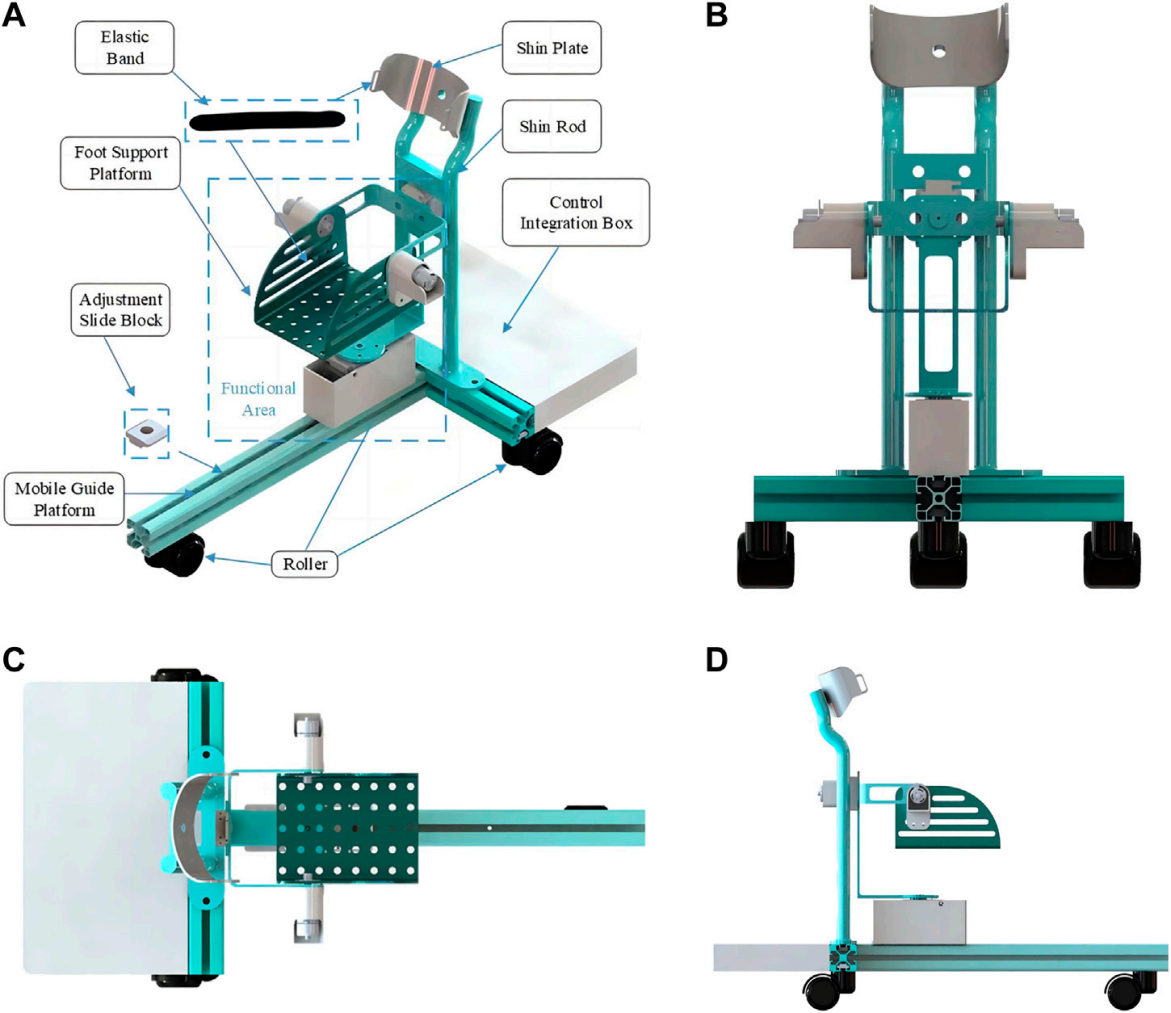
RARR model diagram. **(A)** Installation schematic. **(B)** Front view. **(C)** Top view. **(D)** left view.

The RARR model is illustrated in Figure 1. The structure of RARR comprises several components, including a mobile guide rail platform, adjustable sliders, a shin rod, a shin support plate, a foot support platform, elastic bands, a control integration box, transmission mechanisms, and three non-powered rollers. The three rollers are situated beneath the mobile guide rail platform and facilitate the robot’s movement. These rollers can also be locked to prevent unintended movements during use. During rehabilitation training, the user’s lower leg is secured with elastic bands, isolating the ankle from other lower limb joints to prevent compensatory movements and reduce the load on the robot. The functional sections of RARR are fixed to the mobile guide rail platform using sliders and are driven by motors to facilitate user foot movement during rehabilitation. Mechanical limit structures are designed at each rotational joint to ensure user safety. The control integration box houses the necessary components for the control system. Table 1 provides the mechanical specifications of RARR, detailing the range of motion for each joint (Sun et al., 2019).

**TABLE 1:** Range of motion for RARR.

Motion	Angle range (°)
Internal	0–20
External	0–30
Dorsiflexion	0–30
Plantarflexion	0–50
Inversion	0–40
Eversion	0–30

#### 2.1.1 Reconfigurable design

**FIGURE 2:**
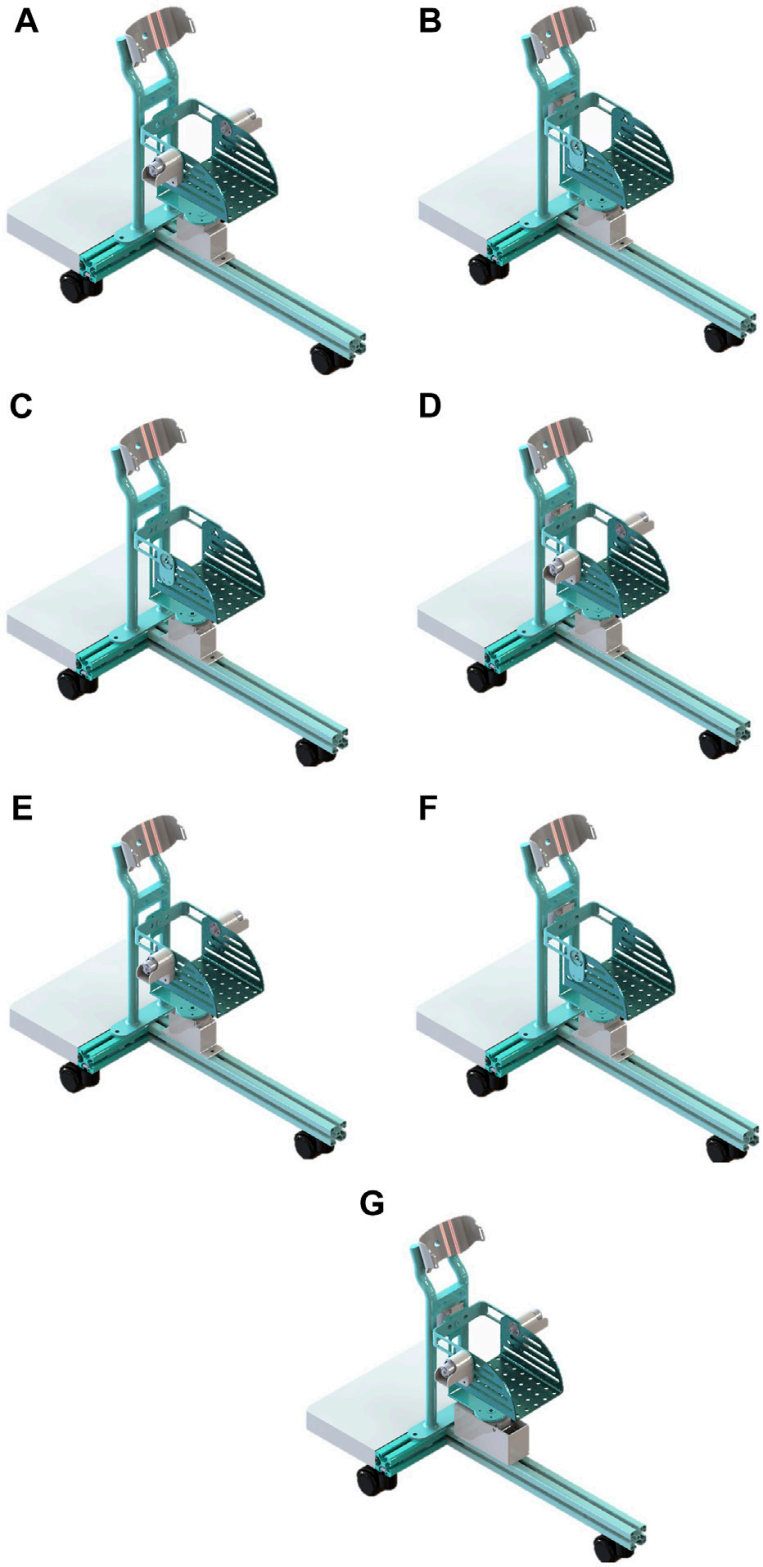
Different modes of RARR. **(A)** Dorsiflexion/plantarflexion. **(B)** Inversion/eversion. **(C)** Internal/external rotation. **(D)** dorsiflexion/plantarflexion with inversion/eversion. **(E)** dorsiflexion/plantarflexion with internal/external rotation. **(F)** Inversion/eversion with internal/external rotation. **(G)** 3-degree-of-Freedom.

In the process of rehabilitation training, multifunctional or reconfigurable robots are more appealing to patients as they can reduce healthcare costs and improve the effectiveness of rehabilitation training (Wang et al., 2022). To cater to the diverse rehabilitation needs of different users, RARR has been designed with reconfigurability, offering an adjustable workspace. By choosing varying numbers of actuators and different configurations for the functional sections, RARR can be reconfigured into 7 different modes, corresponding to 3 single-degree-of-freedom motions, 3 dual-degree-of-freedom motions, and 1 three-degree-of-freedom motion, as depicted in Figure 2.

#### 2.1.2 Adjustable design

**FIGURE 3:**
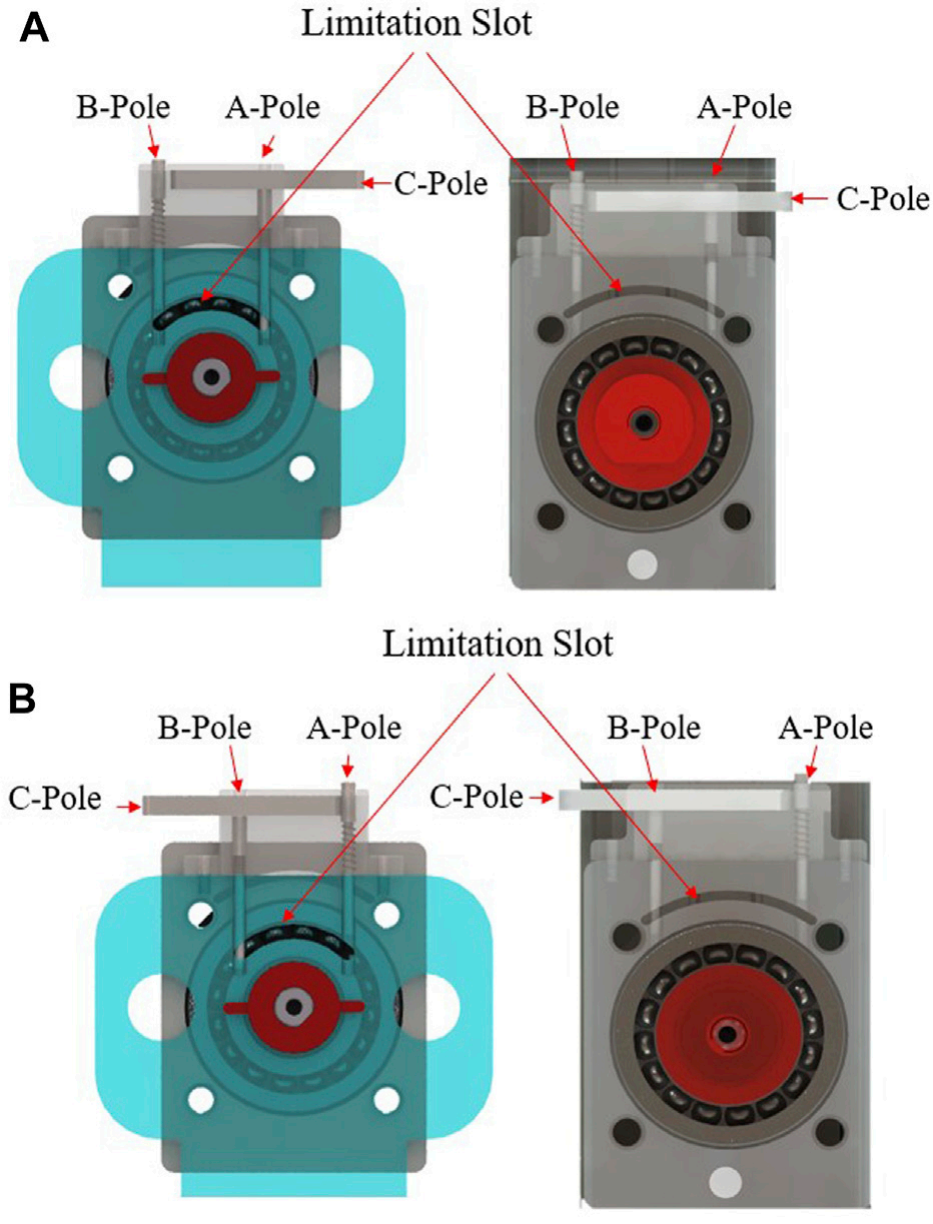
Mechanical limitation at rotational joints. **(A)** Limitation device for left foot rehabilitation training. **(B)** Mechanical limitation device for right foot rehabilitation training.

To accommodate users with varying lower leg lengths, the robot can be adjusted. By changing the position of the sliders, the functional sections of the robot can be adjusted forward and backwards along the guide rail to suit users of different heights. In RARR, adjustable limit structures are employed at the internal/external rotation and inversion/eversion rotational joints, allowing patients to switch between left and right foot rehabilitation training, as depicted in Figure 3. During left foot rehabilitation training, the user depresses Rod A, pushing its lower end into the limit slot. Simultaneously, Rod C is pushed to the left, securing Rod A. When right foot rehabilitation training is required, Rod B is depressed, pushing its lower end into the limit slot. Rod C is also pushed to the left, securing Rod B. This action simultaneously releases Rod A, which, under the force of a spring, pops out of the limit slot. This adjustment allows for different robot workspaces, facilitating the transition between left and right foot rehabilitation training.

#### 2.1.3 Rotation center matching design

**FIGURE 4:**
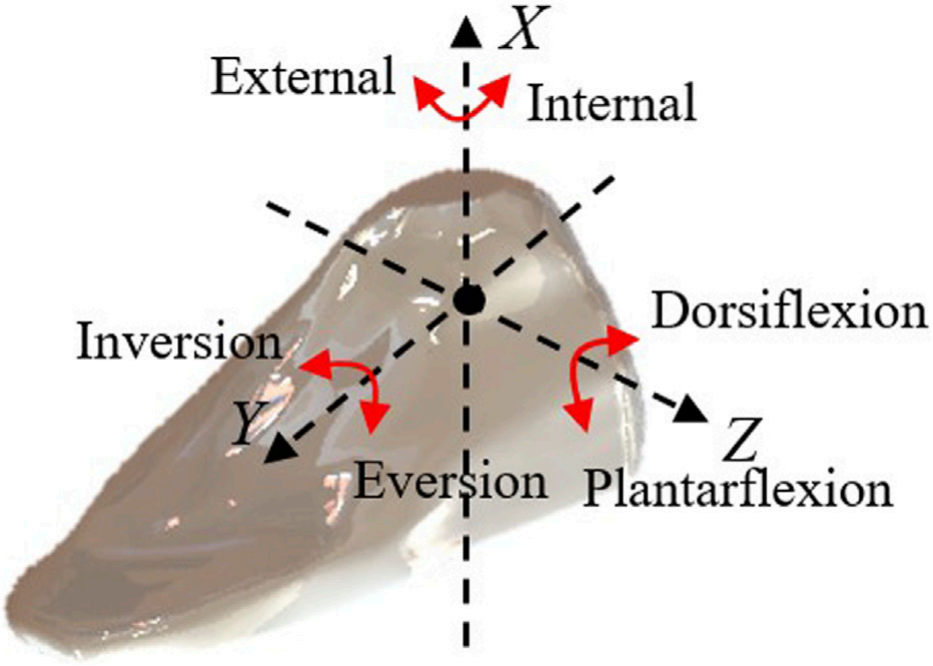
Different directions of ankle joint movement.

**FIGURE 5:**
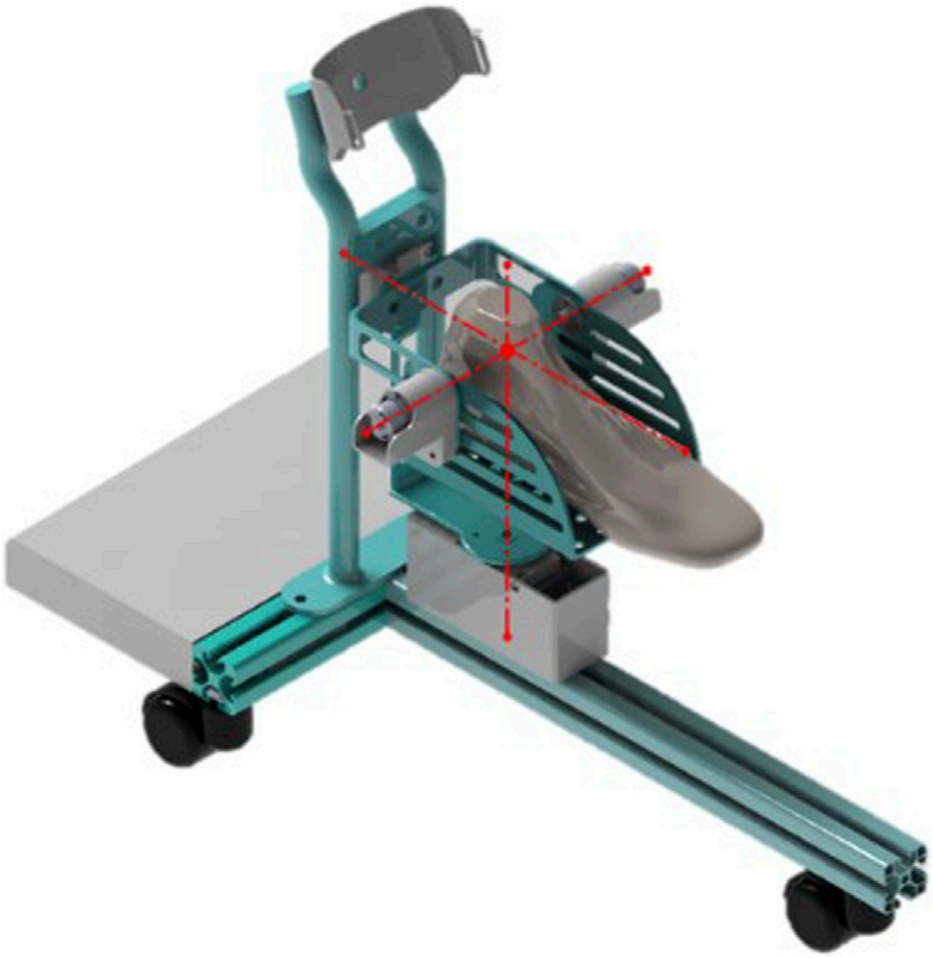
Matching rotation centers.

An important consideration in the design of rehabilitation robots is ensuring that the robot’s rotation center aligns with the ankle joint’s rotation center. Mismatched rotation centers can lead to patient discomfort or even secondary injuries, it is significant to figure out the issue of axis alignment of human-robot coupling (Wang et al., 2022; Cao et al., 2023). Ensuring that the mechanical rotation center of the rehabilitation robot coincides with the ankle joint’s rotation center is a crucial issue in the design process. We achieve this objective through the following methods: 1. Anatomical Research: Anatomical studies and measurements of the human ankle joint are conducted to determine the ankle joint’s axis of rotation and the location of its rotation center, as depicted in Figure 4. This data serves as a reference for robot design to ensure that the robot’s rotation center closely matches or coincides with the ankle joint’s rotation center. 2. Software Simulation: Using relevant software, a human body model is imported, and simulations are conducted to represent the robot’s operation. By comparing the position of the robot’s rotation center with that of the human ankle joint’s rotation center, adjustments are made to optimize the robot’s design parameters to achieve a matching rotation center, as illustrated in Figure 5.

### 2.2 Control design

**FIGURE 6:**
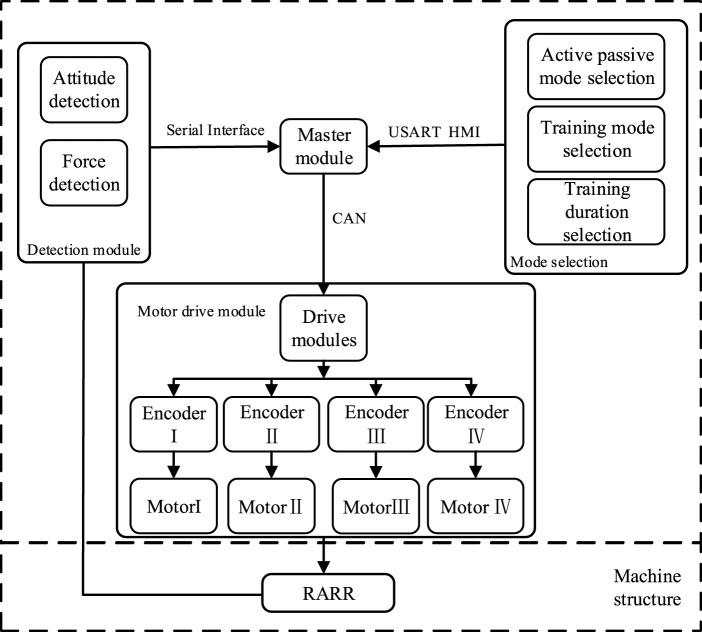
Overall control design of the RARR system.

**FIGURE 7:**
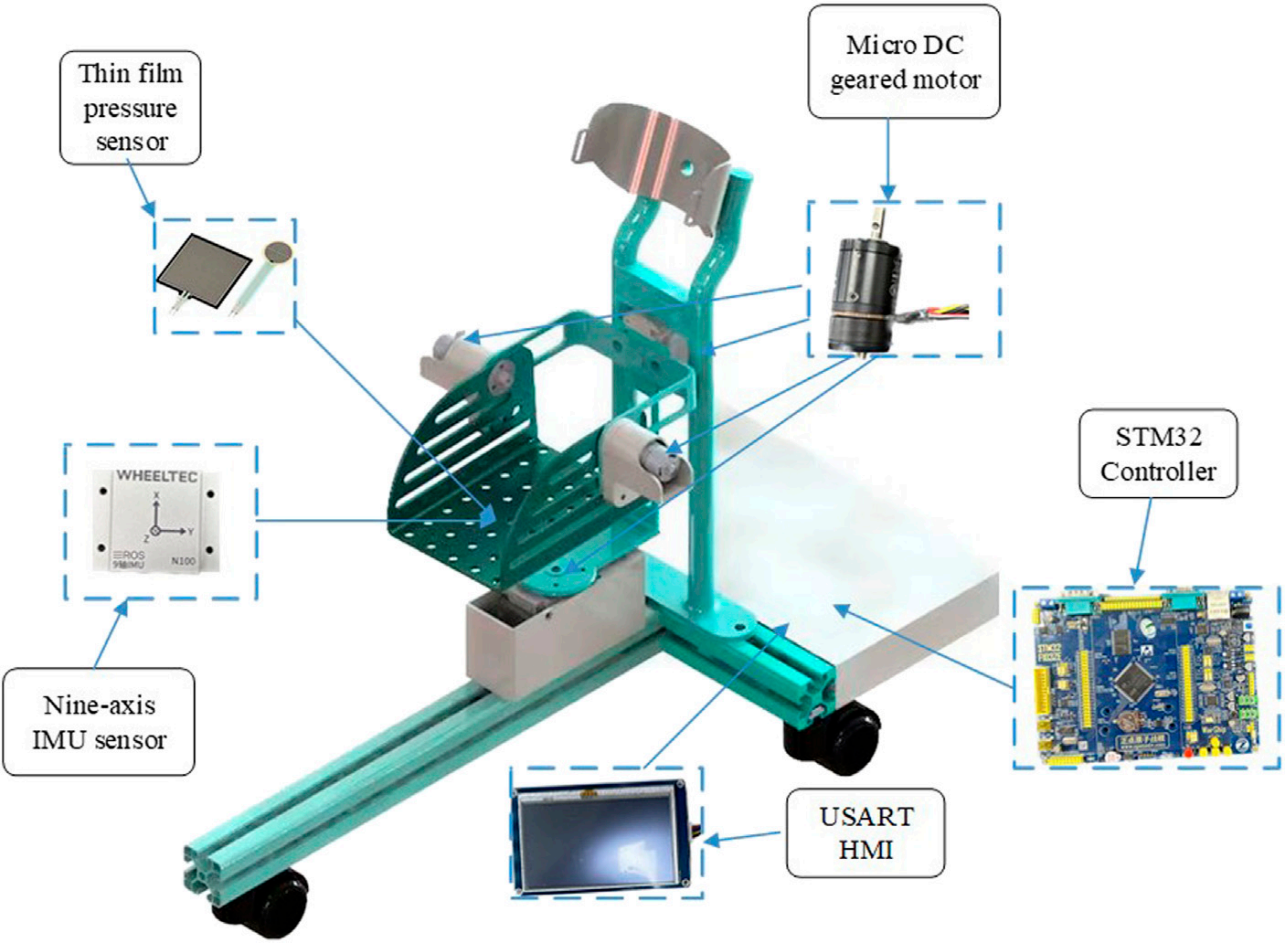
Hardware installation model of RARR.

The RARR control system consists of four main modules: the central control module, sensing module, selection module, and actuation module, as depicted in Figure 6. The central control module utilizes an STM32 controller as the control core, receiving commands and translating them into corresponding signals. The sensing module comprises a nine-axis IMU sensor (N100, WHEELTEC, Dongguan, China, with an angular accuracy of 0.1RMS) and thin-film pressure sensors. This module collects user foot angle information and pressure data, forming the basis for force feedback and position feedback. The selection module is primarily implemented through USART HMI, model TJC4827T043_011, which provides users with a graphical interface. It uses different key values to set motion parameters, achieve the desired goals and complete various training tasks. The actuation module is composed of four brushless DC gear motors and encoders. The controller controls the motion of these four brushless DC motors via the CAN bus, enabling rehabilitation training actions. Simultaneously, the encoders and the sensing module continuously monitor the user’s condition to observe the user’s current information, as shown in Figure 7.

In the course of a patient’s rehabilitation exercise, the controller governs the motion based on received signals and control algorithms. Subsequently, the actual joint angles of the robot are fed back to the central control module through motor encoders and posture sensors, forming a closed-loop control system. This system enables real-time and continuous monitoring and adjustment of the patient’s rehabilitation status.

## 3 Theoretical analysis

### 3.1 Kinematic model

**FIGURE 8:**
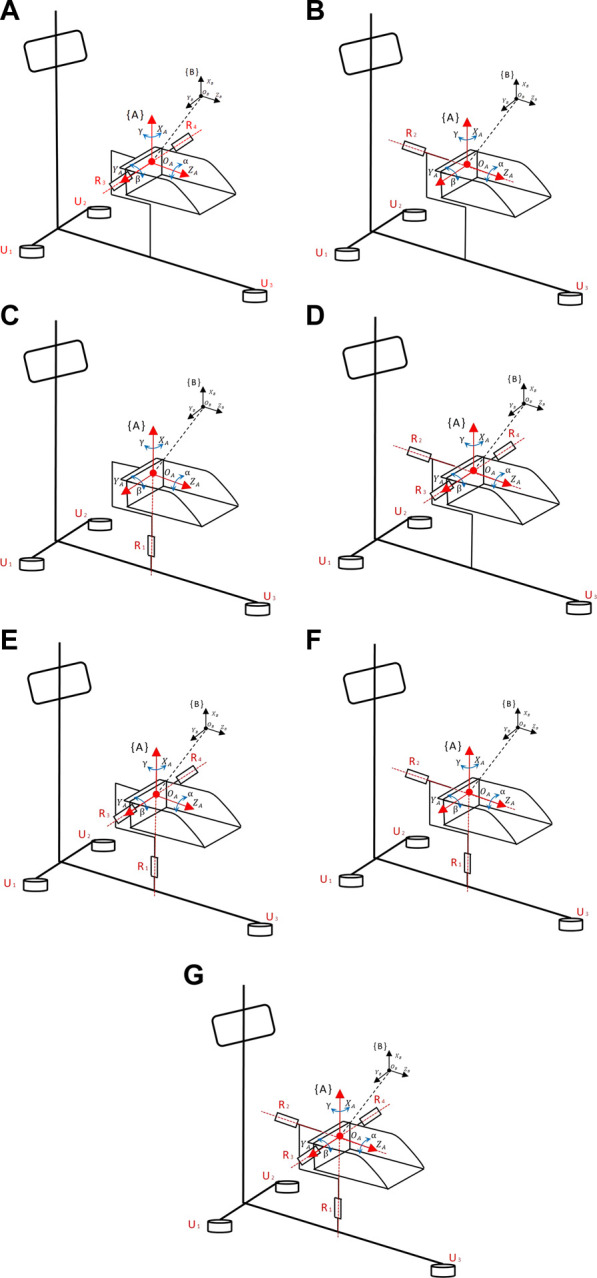
Coordinate diagram of RARR motion structure. **(A)** Dorsiflexion/plantarflexion. **(B)** Inversion/eversion. **(C)** Internal/external rotation. **(D)** dorsiflexion/plantarflexion with inversion/eversion. **(E)** dorsiflexion/plantarflexion with internal/external rotation. **(F)** Inversion/eversion with internal/external rotation. **(G)** 3-degree-of-Freedom.

**FIGURE 9:**
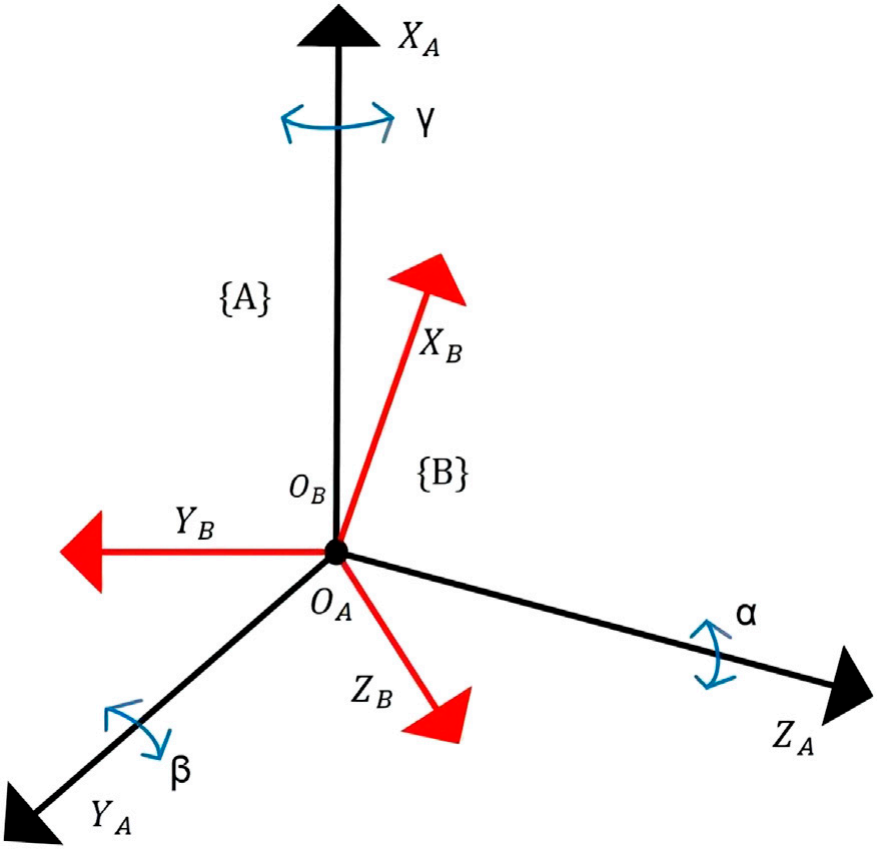
Simplified coordinate diagram of RARR.

The kinematic analysis of rehabilitation robots is of significant importance for workspace analysis, motion trajectory planning, and the determination of the robot’s feasibility. Therefore, it is necessary to establish a kinematic model for the analysis of relevant kinematics (Meng et al., 2023a). In the robot’s motion, a Cartesian coordinate system is established using ***RPY*** angles. Initially, a stationary coordinate system **{A}** is set at the robot’s virtual rest center as a reference frame. Its *Z*-axis is parallel to the footplate plane, pointing toward the far end of the footplate, the *X*-axis is perpendicular to the footplate plane, pointing upward, and the *Y*-axis is parallel to the footplate plane, pointing to the right of the footplate. Simultaneously, a moving coordinate system **{B}** is established at the same origin, moving with the platform, with its axes oriented similarly to coordinate system **{A}**, representing the post-motion footplate plane, as shown in Figure 8 and simplified in Figure 9. The relationship between coordinate system **B** relative to coordinate system **A** can describe the state of an object.

#### 3.1.1 Forward kinematic analysis

Here, ***γ, β*** and ***α*** represent the rotation angles of coordinate system **B** around $X_{A},Y_{A}$ and $Z_{A}$ , respectively. The rotation matrix represented using ***RPY*** angles is as follows:

RXYZBAγ,β,α=RZαRYβRXγ=cα−sα0sαcα0001cβ0sβ010−sβ0cβ1000cγ−sγ0sγcγ=cαcβcαsβsγ−sαcγcαsβcγ+sαsγsαcβsαsβsγ+cαcγsαsβcγ−cαsγ−sβcβsγcβcγ (1)

When performing dorsiflexion/plantarflexion motion, coordinate system **{B}** rotates around $X_{A}$ and $Z_{A}$ angles are 0, which means α=0 and γ=0 . Therefore, its kinematic equation is as follows:

RXYZBAγ,β,α=RZαRYβRXγ=100010001cβ0sβ010−sβ0cβ100010001=cβ0sβ010−sβ0cβ (2)

Similarly, we can derive the kinematic models for the other modes as follows:

Inversion/Eversion Motion Mode:

RXYZBAγ,β,α=cα−sα0sαcα0001 (3)

Internal/External Rotation Motion Mode:

RXYZBAγ,β,α=1000cγ−sγ0sγcγ (4)

Dorsiflexion/Plantarflexion and Inversion/Eversion Motion Mode:

RXYZBAγ,β,α=cαcβ−sαcαsβsαcβcαsαsβ−sβ0cβ (5)

Dorsiflexion/Plantarflexion and Internal/External Rotation Motion Mode:

RXYZBAγ,β,α=cβsβsγsβcγ0cγ−sγ−sβcβsγcβcγ (6)

Inversion/Eversion and Internal/External Rotation Motion Mode:

RXYZBAγ,β,α=cα−sαcγsαsγsαcαcγ−cαsγ0sγcγ (7)

Three Degrees of Freedom Motion Mode:

RXYZBAγ,β,α=cαcβcαsβsγ−sαcγcαsβcγ+sαsγsαcβsαsβsγ+cαcγsαsβcγ−cαsγ−sβcβsγcβcγ (8)

#### 3.1.2 Inverse kinematic analysis

Given the rotation matrix, we can deduce the **XYZ** fixed-angle representation in ***RPY***. Let:

RXYZBAγ,β,α=r11r12r13r21r22r23r31r32r33=cαcβcαsβsγ−sαcγcαsβcγ+sαsγsαcβsαsβsγ+cαcγsαsβcγ−cαsγ−sβcβsγcβcγ (9)

Through derivation, we can obtain:

$$c\beta =\pm \sqrt{r_{11}^{2}+r_{21}^{2}}$$ (10)

By dividing $-r_{31}$ by cβ , we obtain tan⁡β , and finally, taking the arctan will give us:

$$\beta =A\tan2\left(-r_{31},\sqrt{r_{11}^{2}+r_{21}^{2}}\right)$$ (11)

Based on the normal range of motion of the human ankle joint and the mechanical limit design of the robot, we can determine that $-50^{。}\leq \beta \leq 30^{。}$ and cβ≠0, thus:

$$\alpha =A\tan2\left(r_{21},r_{11}\right),\beta =A\tan2\left(-r_{31},\sqrt{r_{11}^{2}+r_{21}^{2}}\right),\gamma =A\tan2\left(r_{32},r_{33}\right)$$ (12)

Where $A\tan2\left(y,x\right)$ is the two-argument arctangent function, with a range of values in $\left[-\pi ,\pi \right]$ .

### 3.2 Workspace analysis

**FIGURE 10:**
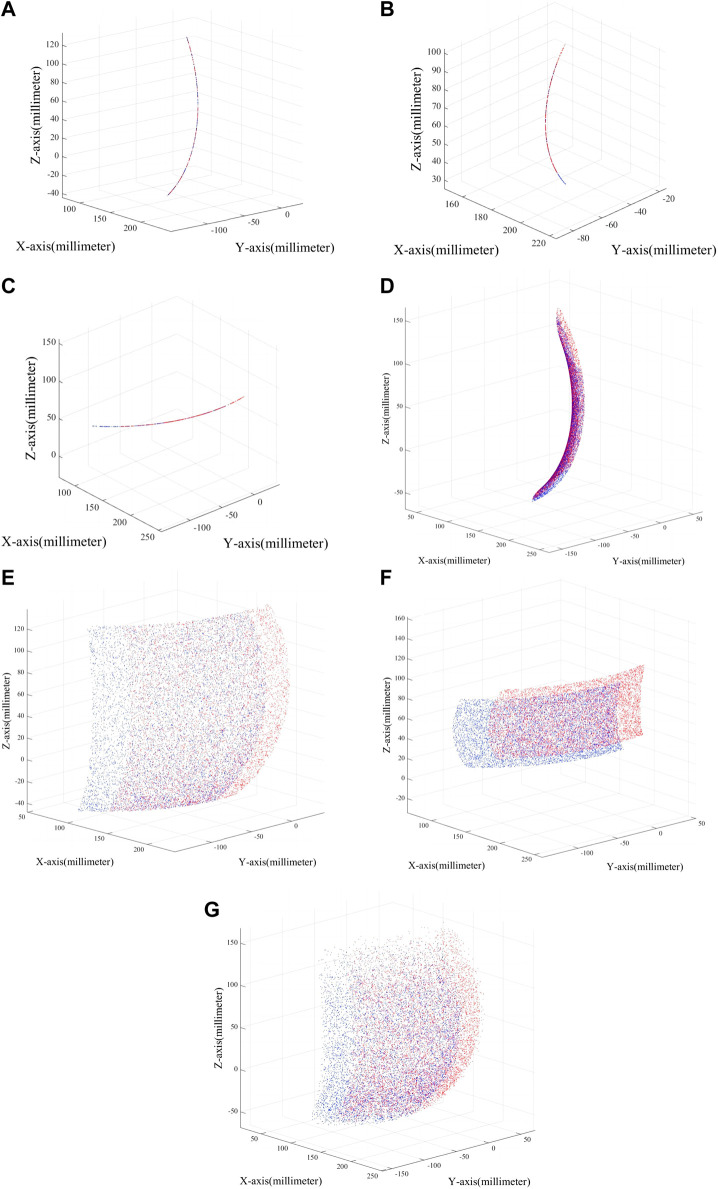
Robot workspace in different modes. **(A)** Dorsiflexion/plantarflexion. **(B)** Inversion/eversion. **(C)** Internal/external rotation. **(D)** dorsiflexion/plantarflexion with inversion/eversion. **(E)** dorsiflexion/plantarflexion with internal/external rotation. **(F)** Inversion/eversion with internal/external rotation. **(G)** 3-degree-of-Freedom.

The workspace of the robot is a crucial metric for evaluating its feasibility, as it reflects the robot’s performance and directly impacts its practical application value (Meng et al., 2023b). To visually represent the variability of RARR’s workspace, based on the kinematic model of the ankle rehabilitation robot, a Monte Carlo random sampling method was used to plot a large number of end-effector positions to achieve visualization of the robot’s workspace, as shown in Figure 10.

Figure 10 displays the variation in the robot’s workspace under different modes. From the figure, it is evident that the robot’s workspace varies with different configurations, and adjustments within the same mode can also lead to different workspaces. Additionally, the robot’s workspace aligns with the physiological parameters of the human ankle joint, making RARR suitable for assisting ankle joint rehabilitation training. The blue area in the figure represents the workspace when using the right foot, while the red area represents the workspace when using the left foot.

### 3.3 Finite element strength verification of key components

One of the primary objectives of this study is to reduce the volume and overall weight of the ankle rehabilitation robot by simplifying the mechanical structure and optimizing materials. This approach aims to minimize psychological stress on patients during the training process and reduce energy consumption. Considering factors such as material yield strength and mass density, 6,061 aluminum alloy was chosen as the primary material for key components of the RARR. The main material properties of 6,061 aluminum alloy are outlined in Table 2. The remaining components are manufactured using 3D printing with photosensitive resin as the material.

**TABLE 2:** Material properties of 6,061 aluminum alloy.

Yield Strength (N/m^2^)	Tensile Strength (N/m^2^)	Young’s Modulus (N/m^2^)	Poisson’s ratio	Mass density (kg/m^3^)	Shear modulus (N/m^2^)
5.51485e+007	1.24084e+008	6.9e+010	0.33	2,700	2.6e+010

**FIGURE 11:**
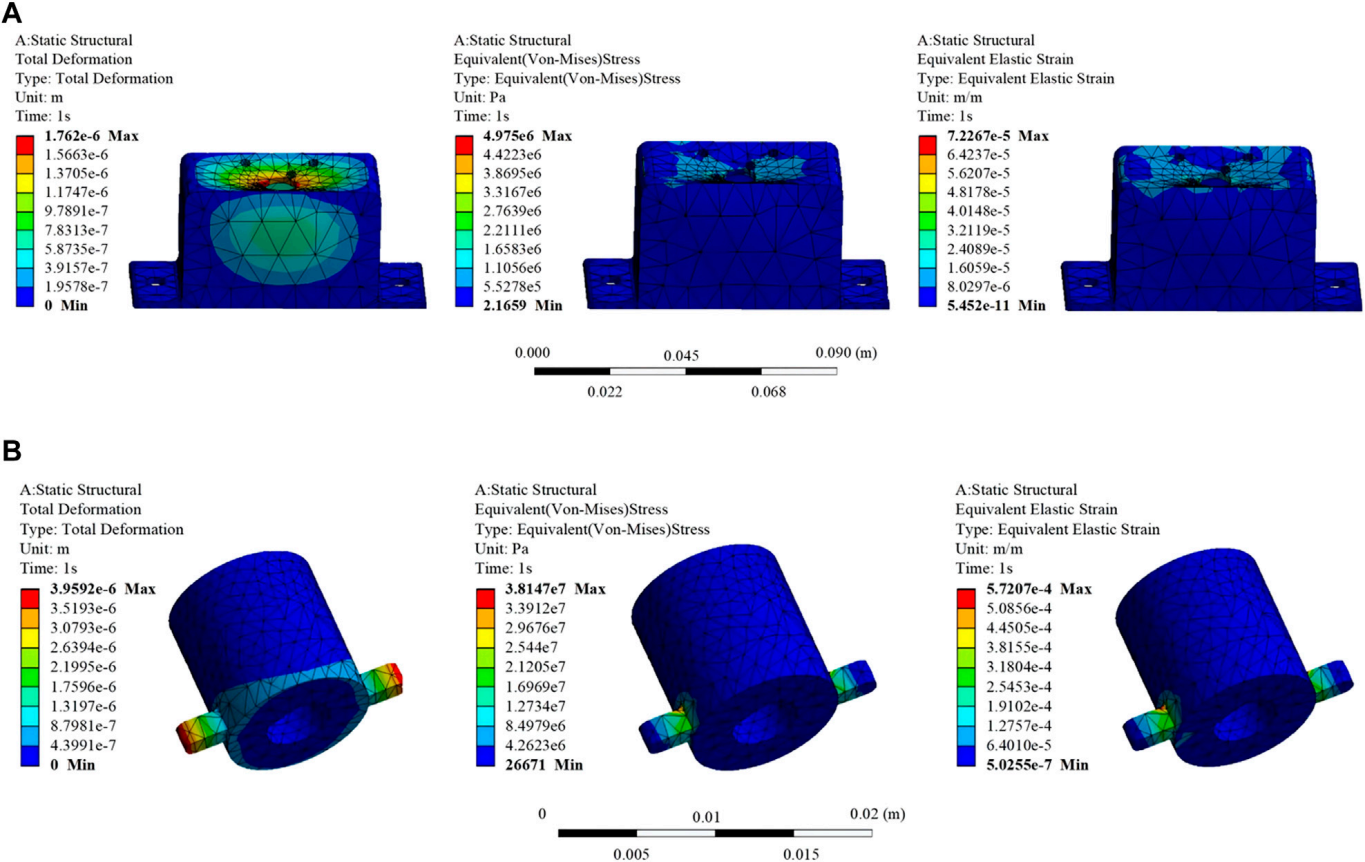
Finite element analysis results of selected key components. **(A)** Part 1: Total deformation, equivalent stress, and equivalent elastic strain results. **(B)** Part 2: Total deformation, equivalent stress, and equivalent elastic strain results.

To validate the reliability and rationality of the mechanical design and material selection in this study, finite element analysis was conducted on selected key components of the designed mechanism, as illustrated in Figure 11.

From the information presented in the figures, it is evident that the maximum stresses locally experienced by Part 1 and Part 2 are 4.975e+006N/m^2^ and 3.8147e+007N/m^2^, respectively. These values are significantly below the material’s yield strength of 5.51485e+007N/m^2^. The maximum strains are 7.2267e-005 m/m and 5.7207e-004 m/m for Part 1 and Part 2, respectively. Additionally, the maximum displacement values are 1.762e-006 and 3.9592e-006 m for Part 1 and Part 2, respectively, indicating minimal deformation. These results suggest that the chosen materials for Key Components 1 and 2 are appropriate, and the dimensional design of the components meets the design requirements.

## 4 Experimental findings

### 4.1 Manufacturing of the prototype and verification of reconfigurability

**FIGURE 12:**
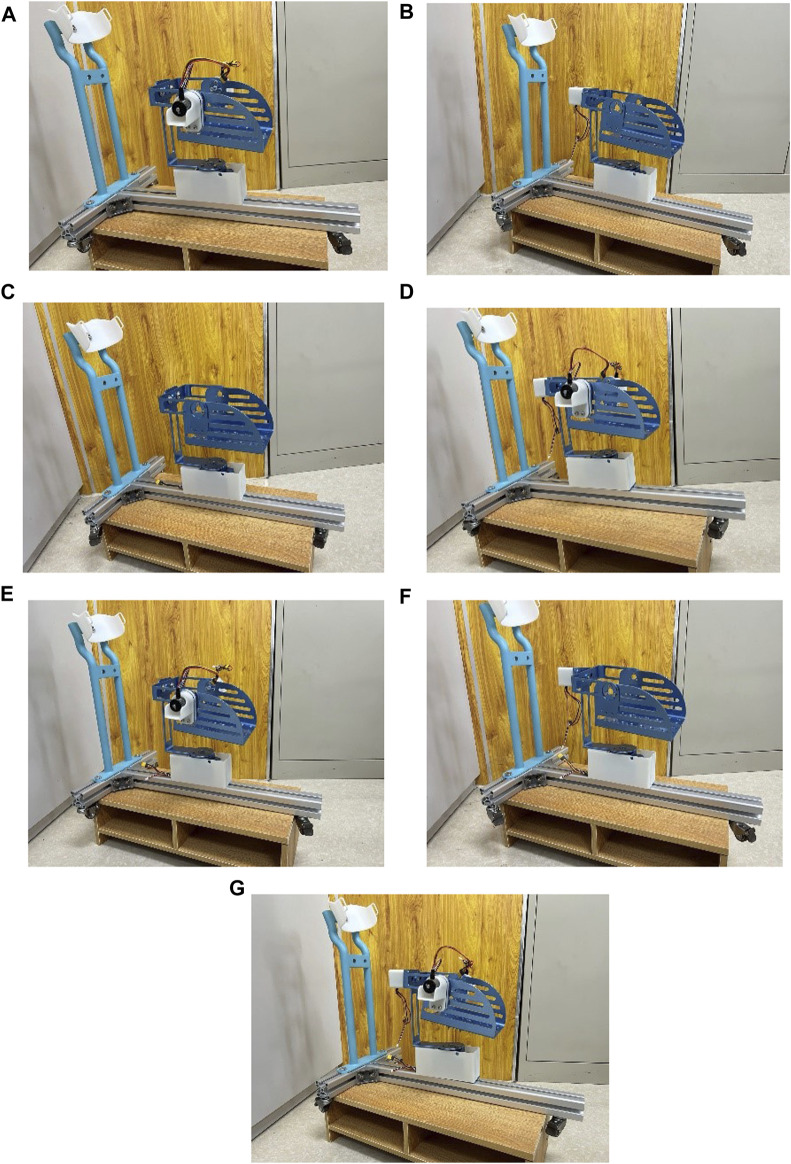
Robot prototype in various assembly modes. **(A)** Dorsiflexion/plantarflexion. **(B)** Inversion/eversion. **(C)** Internal/external rotation. **(D)** dorsiflexion/plantarflexion with inversion/eversion. **(E)** dorsiflexion/plantarflexion with internal/external rotation. **(F)** Inversion/eversion with internal/external rotation. **(G)** 3-degree-of-Freedom.

In accordance with the design plan, the prototype was successfully manufactured, and seven different assembly modes were realized through various assembly methods, as illustrated in Figure 12. Importantly, there were no interferences during the robot assembly process. The successful manufacturing of the experimental prototype validates the rationality and reconfigurability of the robot design.

### 4.2 Passive control rehabilitation training experiment

One healthy subject (male, 26 years old, height 178 cm, weight 60 kg) was recruited to undergo passive rehabilitation training. This study was conducted with the approval of the Ethics Review Committee of Shanghai University of Medicine and Health Sciences (Approval Number: 2022-zyxm2-04--420300197109053525), and all procedures adhered to the standards outlined in the Helsinki Declaration.

**FIGURE 13:**
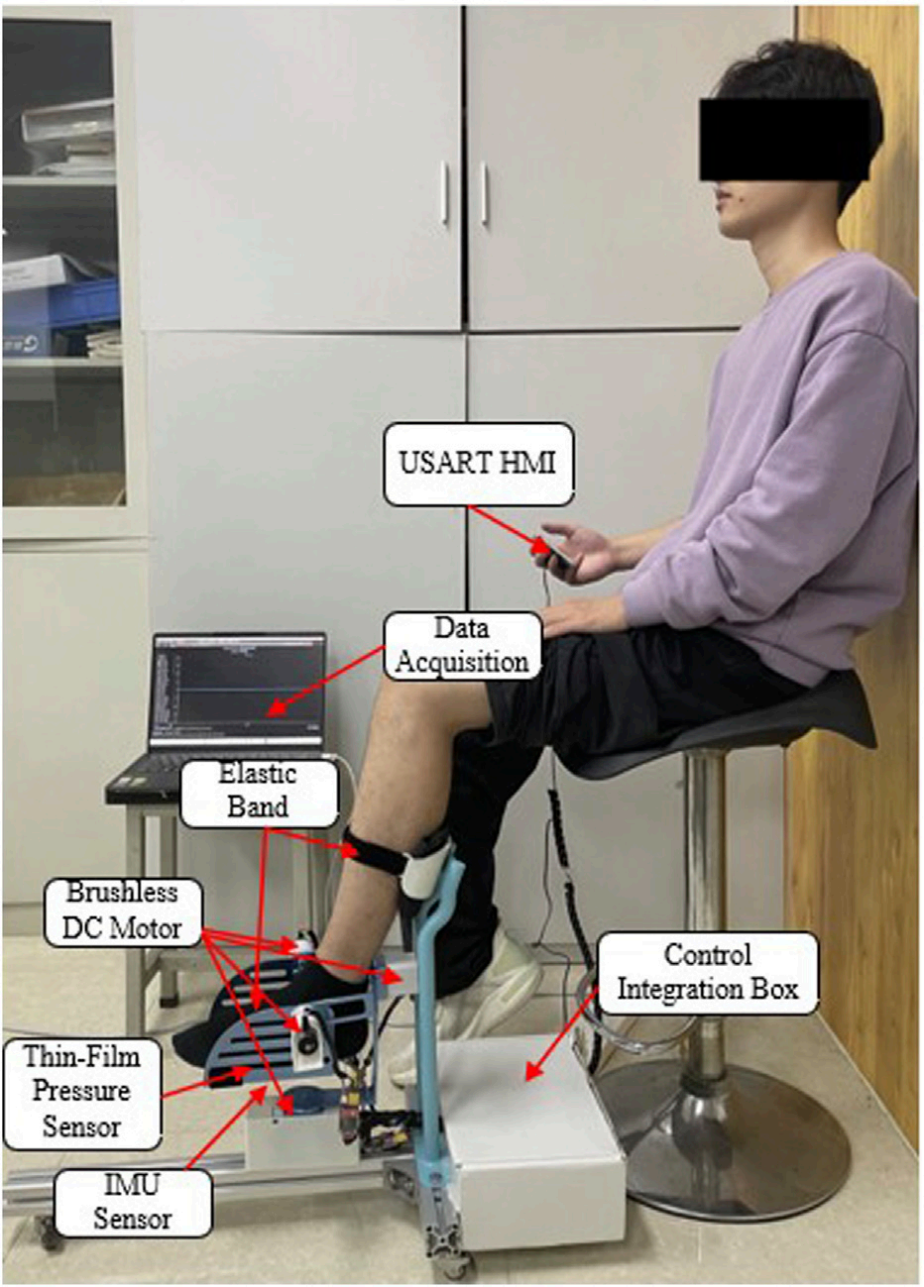
Passive control rehabilitation training experiment.

Passive rehabilitation training is suitable for early rehabilitation of ankle joint dysfunction patients who may exhibit symptoms of muscle weakness, low muscle strength, or high muscle tone. Hence, the experimental setup involved lower joint operating speeds to ensure system safety. After donning the experimental prototype, the subject underwent passive control rehabilitation training to verify the effectiveness and stability of joint motion during the testing, as shown in Figure 13.

**FIGURE 14:**
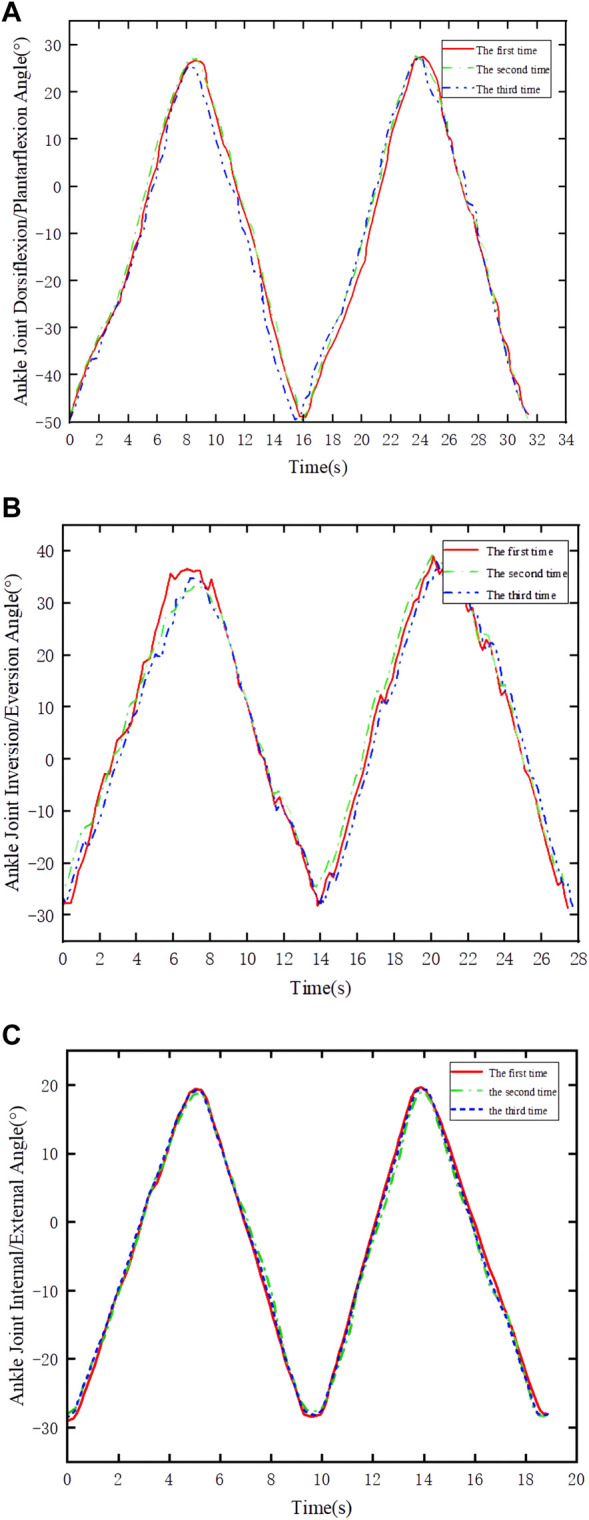
Joint angle variations in passive control experiments. **(A)** Dorsiflexion/plantarflexion. **(B)** Inversion/eversion. **(C)** Internal rotation/external rotation.

During the training process, first, zeroing the IMU device was performed to address errors caused by the installation of the IMU by the experimenter (Yang et al., 2023). Subsequently, a nine-axis IMU sensor was utilized to monitor the patient’s posture and acquire the robot’s motion performance. Three experiments were conducted with the same motion, as depicted in Figure 14. The experimental results indicate that the training trajectory during passive rehabilitation is smooth, without abrupt angle changes. However, due to interference errors during operation, some angles exhibit fluctuations. In summary, the RARR, driven by direct motor control, moves the footplate to enable ankle rehabilitation training, thereby meeting the user’s needs for strengthening ankle muscle, restoring joint mobility, and enhancing ankle joint stability. This robot fulfills its design purpose for assisting in rehabilitation training.

## 5 Conclusion and future prospects

This study has successfully developed and evaluated a multi-degree-of-freedom, reconfigurable ankle rehabilitation robot with a variable working space to assist in the rehabilitation training of patients with ankle dysfunction following a stroke. The robot’s variable working space and reconfigurable characteristics allow it to adapt to seven different modes, catering to the diverse rehabilitation needs of users. The mechanism incorporates three rotational degrees of freedom around the human ankle’s rotation center, reducing discomfort for users during operation. Theoretical analysis was conducted to determine the relevant parameters of the robot and validate its performance. Subsequent experiments demonstrated the robot’s reconfigurability and the smoothness of training trajectories, aligning with the design objectives. Designed with principles of human factors engineering, the robot features a compact structure, portability, and cost-effectiveness, making it suitable for home use by patients. The modular design helps reduce equipment usage and maintenance costs, depending on the robot’s assembly mode, its manufacturing cost ranges from $800 to $1200. As a result, the developed Reconfigurable Ankle Rehabilitation Robot (RARR) holds practical application value in ankle rehabilitation and is primed for widespread use. Furthermore, we believe that the design methods/principles of this device can provide valuable insights for the design of devices for other body parts. For example, in the design of upper limb exoskeleton robots, the interchangeable and adjustable design can facilitate patients in performing rehabilitation exercises for different limbs. Additionally, the adjustability of robot mechanism lengths can accommodate various patients. The application of a simple, compact, and portable design concept not only reduces the production cost of the robot but also allows patients to use it in different settings. In the future, we will continue to enhance the device’s practicality and aesthetics, integrating various human-machine interaction modes such as voice control and a plantar electrostimulation system to increase its utility. Clinical trials will also be conducted to further enhance the rehabilitation effectiveness in clinical practice.

## Data Availability

The raw data supporting the conclusion of this article will be made available by the authors, without undue reservation.
